# End of Life Intervention Program During COVID-19 in Vall d'Hebron University Hospital

**DOI:** 10.3389/fpsyt.2021.608973

**Published:** 2021-05-03

**Authors:** Anna Beneria, Eudald Castell-Panisello, Marta Sorribes-Puertas, Mireia Forner-Puntonet, Laia Serrat, Sara García-González, Maria Garriga, Carmen Simon, Consuelo Raya, Maria José Montes, Giuliana Rios, Rosa Bosch, Bárbara Citoler, Helena Closa, Montserrat Corrales, Constanza Daigre, Mercedes Delgado, Maria Emilia Dip, Neus Estelrich, Carlos Jacas, Benjamin Lara, Jorge Lugo-Marin, Zaira Nieto-Fernández, Christina Regales, Pol Ibáñez, Eunice Blanco, Josep Antoni Ramos-Quiroga

**Affiliations:** ^1^Department of Psychiatry, Vall d'Hebron Hospital Universitari, Vall d'Hebron Barcelona Hospital Campus, Barcelona, Spain; ^2^Group of Psychiatry, Mental Health and Addictions, Vall d'Hebron Research Institute, Barcelona, Spain; ^3^Department of Psychiatry and Forensic Medicine, Universitat Autònoma de Barcelona, Barcelona, Spain; ^4^Department of Social Work, Hospital Universitari Vall d'Hebron, Vall d'Hebron Barcelona Hospital Campus, Barcelona, Spain; ^5^Biomedical Network Research Centre on Mental Health (CIBERSAM), Barcelona, Spain

**Keywords:** end of life, intervention program, COVID-19, mental health, grief, prolonged grief disorder, death, mourning

## Abstract

**Introduction:** The coronavirus disease 19 (COVID-19) and its consequences have placed our societies and healthcare systems under pressure. Also, a major impact on the individual and societal experience of death, dying, and bereavement has been observed. Factors such as social distancing, unexpected death or not being able to say goodbye, which might predict Prolonged Grief Disorder (PGD), are taking place. Moreover, hospitals have become a habitual place for End of Life (EOL) situations but not in the usual conditions because, for example, mitigation measures prevent families from being together with hospitalized relatives. Therefore, we implemented an EOL program with a multidisciplinary team involving health social workers (HSW) and clinical psychologists (CP) in coordination with the medical teams and nursing staff.

**Objectives:** We aim to describe an EOL intervention program implemented during COVID-19 in the Vall d'Hebron University Hospital (HUVH). We present its structure, circuit, and functions. Descriptive analyses of the sample and the interventions that required psychological and social attention are reported.

**Material and methods:** The total sample consists of 359 relatives of 219 EOL patients. Inclusion criteria were families cared for during the COVID-19 pandemic with family patients admitted to the HUVH in an EOL situation regardless of whether or not the patient was diagnosed with COVID-19.

**Results:** Our program is based on family EOL care perceptions and the COVID-19 context features that hinder EOL situations. The program attended 219 families, of which 55.3% were COVID-19 patients and 44.7% had other pathologies. The EOL intervention program was activated in most of the EOL situations, specifically, in 85% of cases, and 78% of relatives were able to come and say goodbye to their loved ones. An emotional impact on the EOL team was reported. It is necessary to dignify the EOL situation in the COVID-19 pandemic, and appropriate psychosocial attention is needed to try to minimize future complications in grief processes and mitigate PGD.

## Introduction

On March 11, 2020, the World Health Organization (1) declared the severe acute respiratory syndrome coronavirus 2 (SARS-CoV-2 or COVID-19) outbreak a pandemic disease. In Spain, the first case of COVID-19 was diagnosed on January 29 (2). On March 14, the Spanish Government declared a national emergency, which implied the imposition of quarantine on the entire population (3). Such mitigation measures clearly helped contain the disease and flatten the curve (4) but they had an impact on psychological health (5) and important socio-economic repercussions (6).

The pandemic has seriously challenged our national healthcare system (7), as well as the professionals' mental health (8). One of the key features of COVID-19 is its severity, with a mortality rate around 5.7% (9). To date, more than 100 million cases of COVID-19 have been confirmed, and 2,455,331 deaths have been registered (10). Therefore, death has been around more than ever before.

Death and grief are universal, inevitable, and multidimensional experiences and imply losses which can occur at any stage of life (11). The grieving process reflects a unique convergence of responses (affective, cognitive, behavioral, physiological, and spiritual adjustments) which affect both the individual (12) and the family system (13). In 2013, the Diagnostic and Statistical Manual of Mental Disorders (DSM-5) (14) proposed Persistent Complex Bereavement Disorder (PCBD) and recently prolonged grief disorder (PGD) was formally included in the 11th revision of the International Classification of Diseases (15). Despite the fact that bereavement is a typically severe stressor that implies painful grief symptoms which can interfere in peoples' lives through higher symptom intensity and/or duration, there is usually no need for clinical intervention (16).

However, it has been observed that COVID-19 conditions might exacerbate the chance of developing PGD (17). Several studied factors associated with PGD occur and challenge the mourning processes (18). From a general perspective, we are aware that PGD is more likely to take place in disasters with many casualties (19). Specific features that increase the chance of PGD appearance include the lack of preparedness or unexpected death, not knowing about the quality of the caregiving or dying experiences, absence of physical social support (20), or grief rituals (e.g., saying goodbye or viewing and burial of the body) (21). Indeed, most recent literature is reporting on bereavement responses during COVID-19. Eisma et al. (22) found more severity in grief reactions after COVID-19-related bereavement compared to natural bereavement (but not unnatural bereavement). Along the same lines, Eisma and Tamminga (23) demonstrated that people who experienced a recent loss during the pandemic had higher grief levels than people who experienced it before the pandemic.

Also closely linked to losses and mourning processes, we would like to focus on End of Life (EOL) situations in the hospitalization context, which have become challenging for families, causing them confusion and distress (24). Partial or total restrictions on relatives' visiting have been imposed, driven by the need to limit spread of the disease (25). Lack of information on the process and inconsistency of the mitigation measures prevent relatives from adapting properly to the situation (24). This might well-affect relatives either with or without family members diagnosed with COVID-19 because government policies applied to all hospitalized people (26). Actually, most recent research reports on the shocking experience of relatives who have lost their loved one in a COVID-19 hospitalization context: being apart during hospitalization and death, cold communication of bad news, lack of social support and death rituals, unexpected and fast death or feeling of unfairness (27).

All these features can hinder proper care of EOL situations (28). Managing death and mourning during COVID-19 has become crucial, not only to avoid situations of dying in absolute isolation but also to give patients and relatives the chance to be accompanied in EOL situations (29). Families definitely care about how their relatives depart, and this includes providing the desired physical comfort, emotional support, the possibility to participate in decision-making processes, treating the end with respect, coordinating the care provided, and taking their emotional needs into account (30). This goes together with the desire of the patients in an EOL situation to relieve their relatives' burden and strengthen contact with them (31).

The COVID-19 pandemic has contributed to increasing difficult circumstances and the potential for amplified grief. Therefore, healthcare clinicians need tools and resources to mitigate the grief with which patients and families must cope with. The Psychiatry Department of Vall d'Hebron University Hospital (HUVH) identified the need to develop an “EOL intervention program” formed by clinical psychologists (CP) and healthcare social workers (HSW) that provided face-to-face support to relatives in EOL situations. We aim to describe the structure, circuit, and functions of the intervention program, and also to analyze which type of sample and interventions required psychological and social attention.

## Context

### Socio-Demographic Characteristics of the Population in the HUVH

HUVH is located in the north of Barcelona, within the Horta-Guinardó district. It is a public hospital of the Hospital Network of the Catalan Health Institute and is a referral center in the comprehensive healthcare area of the north of the city, which includes three districts, Horta-Guinardó, Nou Barris, Sant Andreu, and the city of Montcada i Reixac, with a total population of more than 480,000 inhabitants. However, HUVH is a tertiary care hospital, so it receives patients from all over the country.

Among the socio-demographic characteristics of the population that attends the HUVH, we highlight the aging population, which is especially concentrated in the covered areas (47.59% aged over 65) (32, 33). Moreover, Nou Barris is the district of Barcelona with the lowest average annual income per household and person, and has one of the highest percentage of migrants in the city, 17% (32). These socio-demographic characteristics can become risk factors in health-disease processes, and can place them in a situation of high vulnerability and social exclusion (34–37). In the context of the pandemic, these social determinants of health have become notorious in the HUVH (38), especially since Nou Barris became the district most affected by COVID-19 in Catalonia during lockdown.

Some specific factors that may increase the population's burden were observed: first, home-isolation difficulties, due to the overcrowding or infra-housing situation, causing the contagion of entire families or cohabitation units; economic-labor fragility situations such as unemployment or submerged economy (39); and finally, migrated families often without a socio-familial network (40). All these factors have converged and caused stress on grief processes and the reorganization of their family systems.

### Setting

One of the main challenges of the pandemic outbreak was the need to transform the activity and capacity of the hospital to attend patients with COVID-19, often withdrawing treatment from other patients who had non-pandemic-related health needs (41).

On March 10, 2020, the contingency plan of the HUVH was approved and subsequently communicated to the staff organization. During the following weeks, the hospital had to reformulate all the spaces progressively, starting with the Emergency Department as well as COVID-19 and non-COVID-19 areas. In this process of transition, the usual 56 intensive care unit (ICU) beds available were multiplied by almost seven. When the epidemic reached its peak, the HUVH had 700 COVID-19 beds, and it was 50 beds away from collapse.

Health professionals had to adapt to the pandemic in a very short time, experiencing changes in their working shift organization, and increasing the burden of care. At the same time, the global surge in demand led to shortages in protection equipment, masks, and other medical devices like respirators. All this exacerbated concerns about the increased risk of infection (8).

In this severe context, national and hospital policies determined whether hospital visits were allowed. In light of COVID-19, much tougher restrictions were established to protect the patients, hospital staff, and visitors (42, 43). Initially, and only for a few days, visits were completely suspended. However, on March 26 visits to patients in EOL situations were allowed. The requirement to attend these families was immediately detected, and from March 30 to April 2, the EOL program was designed, coordinated and structured, starting officially on April 3 and lasting May 31. From May 27, visitors were permitted, with restrictions that were adapted to requirements over time. From then until now, the EOL service has been working, if necessary, with the liaison and inter-consultation unit.

To support those relatives who came to the hospital in tough emotional conditions, it was necessary to reorganize and increase the number of specialized staff delivering EOL care. Initially, the EOL care circuit was attended to by professionals from the Citizen Service Unit of the hospital. Subsequently, due to the families' perceived emotional and psychosocial support needs, social work professionals, together with CP, attended to the EOL processes.

### Key Programmatic Elements: EOL Intervention Program

The EOL Intervention Program arose from the need to attenuate the hospital's environment restrictions during the COVID-19 pandemic (44). HSW and CP assessed the importance of addressing this moment of change to facilitate the mourning process. The aims of the intervention program are based on the importance of a dignified farewell (see Table 1). These experiences will become part of the family's history, and the team considered them a basic rightfor both the dying patient and the family to be able to say goodbye to each other (45).

**Table 1:** Aims of the EOL intervention program.

1	Minimize the impact of the state of alarm produced by the COVID-19 pandemic on families in a hospitalization context How: supporting families in a farewell situation in a hostile context
2	Give the family the chance to say goodbye to their dying loved one and support them during this moment of crisis How: Allow relatives to say farewell to their loved one face to face with all the required protective measures and offer support during this process
3	Care for an individual's mourning process, consequently guiding them toward the most healing path, as well as reducing risks How: Offer psychosocial support during the EOL process and address identified risk factors for PGD development
4	Ease access to clinical psychologists in case of excessive pain, too intense and lasting, which requires specialized attention How: Psychological assessment of the relatives' emotional state, needs and risk factors and consequently refer them to the appropriate public mental health services

*EOL, end of life*.

**Figure 1:**
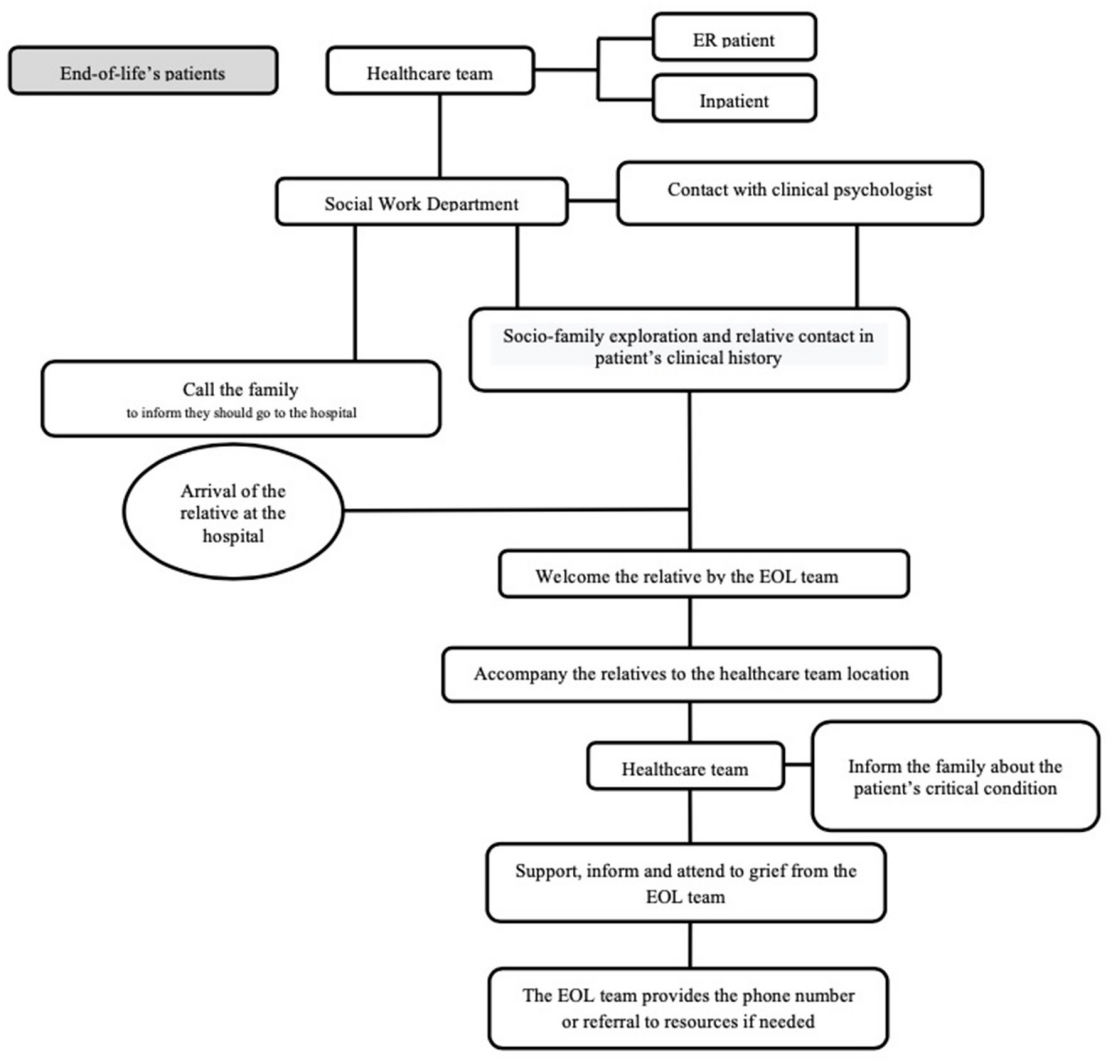
Action circuit for EOL program. ER, emergency room; EOL, End of Life.

Six HSW from the Social Work Department and eighteen CP from the Psychiatry Department (eleven physicians and seven residents) were included in the program. Both teams were trained in psychopathology related to somatic issues, working with integrative theoretical models of reference. Main clinicians' therapy approaches were cognitive-behavioral, systemic, and humanistic. Part of the team was trained in grief counseling, and moreover a few of them belonged to the health consultation-liaison psychology and social work service. Main HSW tasks were to identify social vulnerability settings and needs in order to address them (e.g., inform on funeral procedures, refer to social service centers). CP were able to assess the emotional state and needs of relatives and consequently intervene (e.g., facilitate emotional expression, refer to specialized grief or family support programs). Coordination with healthcare teams (HCT), consisting of doctors and nurses, was required in every attended case. The EOL program covered 24-h shifts every day of the week, and each shift was made up of two professionals from each of the aforementioned categories. The night shift, lasting from 8:00 p.m. to 8:00 a.m., was covered by just one HSW who could contact the Psychiatry Ward if necessary. The action circuit followed in the EOL Intervention Program is presented in Figure 1.

The interventions delivered by the EOL team were divided into two basic kinds of cases: (1) EOL situations (see Table 2) and (2) family interventions to communicate a loss to an inpatient (see Table 3). All the interventions were based on studied factors of EOL family caring such as proper communication, respect, compassion, emotional support, and promotion of emotional expression (46, 47). An essential key of the program was non-intrusive emotional accompaniment, respecting the variability and individuality of reactions and emotional expressions in the face of a crisis (e.g. some needed a safe place to express the sorrow they felt; others required more technical information about funeral procedures). In some cases, this meant accompanying in silence; in others, it consisted of active listening, and in a few the team had to resort to specific techniques for anxiety or crisis intervention. We emphasize the fact that working together as a team seems to have promoted higher coordination and also helped with emotional support among the professionals.

**Table 2:** EOL psychosocial intervention.

**Phases**	**Setting**	**Content**
Activation and coordination	Telematic coordination between HCT and EOL team after detection of an EOL situation.	Basic information gathering - Prognosis of the patient - Number of relatives - What information relatives have - Relatives' contact information
Social assessment	Telematic contact between Social Worker and relatives.	Social assessment - Patient's support network - Main caregivers - Socioeconomic situation Schedule an appointment to come to the hospital
Psychological assessment and pre-intervention	Face-to-face meeting of the EOL team with the relatives in the hospital hall.	First basic psychological assessment - Explore necessity of psychological support - Emotional state - Emotional needs - Psychopathological background, if required
Bad news communication	Face-to-face meeting of the EOL and HCT with the relatives in a private room.	Information of bad news: - Explanation of the evolution - Explanation of the prognosis Psychological support - Promote emotional expression - Promote the expression of doubts and worries regarding the disease course and death - Facilitate the farewell when difficulties facing it
Farewell	Face-to-face and private farewell in the patients' room.	Assure privacy in the farewell momentProvide protection measures to prevent contagion
Post-intervention	Face-to-face meeting of the EOL team with the relatives in a large private room.	Psychological support - Promote emotional expression - Validate experience of loss - Give meaning to the experience - Validate common grief reactions - Promote identified protective factors - Psychoeducate on phases of mourning - Inform about mourning rituals and how to adapt them to COVID-19 context - Advise on how to deliver the news to children - Specific anxiety techniques (e.g., relaxation) if needed Final psychological assessment - Protective factors: social network support, adaptive reactions, anticipation of alternative grief rituals - Risk factors: psychopathological background, high levels of expressed emotion, other symptomatology suggestive of specialized attention Social information: - Funeral services contact and procedures - Bureaucratic aspects - Public aids in socioeconomic risk situations - Specialized contact information: referral to specific resources if needed (e.g., EOL contact, family support program, grief-specific program…)

*EOL, end of life; HCT, health care team*.

**Table 3:** Communication of bad news psychosocial intervention.

**Phases**	**Setting**	**Content**
Activation and coordination	Telematic coordination between HCT and EOL team after detection of the death of an inpatients' loved one.	Coordinate how to manage the information in between patients, HCT and families.
Initial contact	Telematic contact between the EOL team and relatives.	Assessment of needs - Doubts regarding safety issues at the hospital or safepass to come - Doubts about how to deliver the bad news - Emotional state and needs Schedule an appointment to come deliver the bad news
Meeting with the EOL team	Face-to-face meeting of the EOL team with the relative in a large private room.	Psychological support - Promote emotional expression - Normalize associated feelings (e.g., guilt) - Advise on how to deliver the bad news - Information about what reactions to expect - Information about the possibility of psychological intervention with the patient
Bad News Communication	Face-to-face communication of the bad news to the patient by his relative in privacy in the patients' room.	Assure privacy in the delivery of bad newsProvide protection measures to prevent contagionCoordinate with HCT
Post-intervention	Offer to the relative face-to-face meeting with the EOL team.	Psychological supportDebriefing and closure

*EOL, end of life; HCT, health care team*.

We highlight the fact that this entire program was being carried out on the go, and it was modified and improved as regulations in the hospital evolved, and the EOL team faced new problems and needs. For example, at first, there was a strict rule that only one relative could bid farewell to a patient, but as weeks of the pandemic progressed, this was expanded and more relatives were allowed. Also, the location in which to address relatives was problematic, and spaces of every available unit were adapted. Finally, the room for dual pathology and addictions was converted into a temporary room for crisis attention and intervention.

### Caring Team's Experience

The EOL team tried to give the best human and emotional attention to the families coming to bid farewell to their loved ones. We shared painful and sad moments, emotions of love and tenderness, and tough situations with each and every family, at a both personal and professional level. Initially, the team had to retrain in crisis care and mourning, adjust to a new daily reality, with newcare cases, while ensuring that they complied with the safety protocols introduced by the pandemic, such as the use of personal protective equipment. The EOL team was not exempt from a huge emotional impact.

After reviewing the experience with the team, all the EOL professionals expressed this impact, referring to coping with their fears, their work as professionals, wondering whether the program was meeting the needs for which it was created, whether it was worth the trouble, and whether they could help families deal with the pain of their losses. They also mentioned being afraid of their emotions, of reliving the deaths several times a day, of the feelings emerging day by day and having to cope with them during their interventions, of infection and of infecting their relatives. The team also referred to all they had learned, the rewarding aspect of teamwork, the excellent collaboration between coworkers, the need to share with others, to help and be helped, to take care of each other. All the team members appreciate kindness of all the families they helped, who, although undergoing extreme pain, also cared for the professionals who were helping them, and not least, the vast amount of personal health resources human beings have in critical situations.

## Results

After approval by the Clinical Research Ethics Committee (CEIm) of Vall d'Hebron University Hospital PR(AG)435/2020, we analyzed descriptive data collected during the EOL program implementation.

The results obtained from data gathering during the EOL program intervention are shown below. Table 4 shows the socio-demographic data of the patients and their families, describing the main characteristics of the sample. The program attended 359 relatives from a total sample of 219 patients, of which 55.3% were COVID-19 and 44.7% had other pathologies. Mean age of patients in EOL situations was 70.86 (14.85), and 53% were men. As regards relatives, 59.3% were women, and 94.8% had a first-degree relationship, 57.3% being consanguinity related.

**Table 4:** Socio-demographic data of the patients and their families^*^.

	**All Patients**	**COVID-19**	**Other pathologies**
**Patients attended**	*N* = 219	*n* = 121 (55.3%)	*n* = 98 (44.7%)
Men	116 (53%)	58 (47.9%)	58 (59.2%)
Women	103 (47%)	63 (52.1%)	40 (40.8%)
**Mean age**	70.86 (14.85)	71.22 (13.95)	70.42 (15.95)
Men	68.37 (12.54)	67.88 (11.51)	68.86 (13.57)
Women	73.67 (16.71)	74.30 (14.34)	72.68 (18.86)
**Area of origin**			
Barcelona city	169 (77.2%)	105 (86.8%)	64 (65.3%)
Metropolitan area	33 (14.1%)	10 (8.3%)	23 (23.5%)
Other	17 (7.8%)	6 (4.9%)	11 (11.2%)
**Date of hospitalization**			
Before March	9 (4.1%)	6 (5%)	3 (3.1%)
As of March (included)	210 (95.9%)	115 (95%)	95 (96.9%)
Hospitalization time (days)	12.16 (14.35)	16.51 (16.67)	7.37 (9.21)
**Month of intervention**			
Attended to in April	130 (59.4%)	86 (66.2%)	44 (33.8%)
Attended to in May	89 (40.6%)	35 (39.3%)	54 (60.7%)
**Inpatient death**	195 (89%)	103 (85.1%)	92 (93.9%)
**Relatives attended**	359	212 (58.3%)	147 (41.7%)
Men	146 (40.7%)	94 (44.3%)	52 (35.4%)
Women	213 (59.3%)	118 (55.7%)	95 (64.6%)
Average number of relatives attended to	1.76 (1.11)	1.84 (1.18)	1.65 (1)
Relationship degree^*^	*N = 211*		
First-degree^**^	200 (94.8%)	114 (96.6%)	86 (92.5%)
Other	11 (5.2%)	4 (3.4%)	7 (7.5%)
**Relationship**			
Consanguinity	121 (57.3%)	69 (58.5%)	52 (55.9%)
Affinity	31 (14.7%)	12 (10.2%)	19 (20.4%)
Both	59 (28%)	37 (31.4%)	22 (23.7%)
**Families with other members hospitalized**	25	23 (92%)	2 (8%)

* *The data collection was done for a clinical purpose and there's information lost that caused an attrition of eight subjects in some variables*.

** *At least one first-degree relative*.

Concerning the functioning of the program, it was activated in most situations (85%), although in some cases it was not, mostly during the night shift. This was due to communication difficulties because of the chaotic environment and the shift changes between the usual HCT and ward HCT. In general, up to 78% of relatives were able to come and say goodbye to their loved ones. From among the total sample, in one-third of cases (31.8%), the intervention was performed after the death of the patient (see Table 5). This could be explained because the family could not arrive to say goodbye or because the urgency of the situation made it necessary to prioritize the farewell, intervening afterwards.

**Table 5:** Types of intervention.

	**EOL**			**Communication of a death**		
	**COVID-19**	**Other pathologies**	**Total**	**COVID-19**	**Other pathologies**	**Total**
Number of interventions	*n* = 123	*n* = 81	*N =* 204	*n* = 26	*n* = 1	*N* = 27
Case type	54,3%	38,2%	92.5%	7%	0.5%	7.5%
Average interventions by case	1,22 (0.48)	1.14 (0.4)	1.19 (0.45)	2 (1)	1	1.93 (1)
**Intervention type**						
Face-to-face	44.2%	32%	76.2%	50%	7.1%	57.1%
Telephone	14.5%	9.3%	23.8%	42.9%	0%	42.9%
**Shift**						
Day shift	46.5%	24.4%	70.9%	92.9%	7.1%	100%
Night shift	12.2%	16.9%	29.1%	0%	0%	0%
**Localization**						
Hospitalization	22.1%	18.6%	40.7%	28.6%	7.1%	35.7%
ICU	24.4%	11.1%	35.5%	64.3%	0%	64.3%
Emergency department	12.2%	11.6%	23.8%	0%	0%	0%
**Day of intervention**						
Day of death	34.6%	26.5%	61.1%	–	–	–
One day before death	11.1%	6.2%	17.3%	–	–	–
Other	13.6%	8%	21.6%	–	–	–
**Moment of intervention**						
Before death	33.8%	18.9%	52.7%	–	–	–
After death	14.9%	16.9%	31.8%	–	–	–
Both	10.8%	4.7%	15.5%	–	–	–

*ICU, intensive care unit*.

Main interventions were EOL type (92.5%), and in most cases they were performed face to face (76.2%). Despite that, 23.8% were telephone-based, either because they decided not to come or because of illness or other conditions that prevented them from coming. The main reasons were being infected with COVID-19, belonging to a risk group, or living far away in a lockdown context. In a few cases, families reported not wanting to come because of the emotional impact or the emotional distance with their relative. Despite the telematic intervention, the quality of the setting was taken into account, and relatives had full access to psychological and social work aids if necessary. Regarding communication of loss interventions, almost all were performed in COVID-19 cases (96%). It could be explained because this group had substantially more hospitalized relatives, probably because of family clusters transmission.

## Discussion

This study aimed to describe the first face-to-face structured experience of an EOL intervention program during the COVID-19 pandemic. Our experience agrees with the review of Mayland et al. (48), which shows that the pandemic context leads to a disruption, affecting an individual's ability to connect with the deceased both before and after death. This can impact grief, and the usual societal and cultural rituals may appear to be rushed, altered, or absent. Since hospitals became the usual place for EOL situations, and families, especially socio-economically vulnerable ones, were under great stress, it was necessary to develop an EOL program. Our program took all this into account and was based on both specific COVID-19 recommendations (e.g., support to adapt funerals or rituals) (44) and families' perceptions of EOL care (e.g., emotional support) (45).

As expected, (27), due to COVID-19-related restrictions, families with COVID-19 patients as well as families with no COVID-19 patients were affected and benefited from the program, even when the curve was finally beginning to flatten. Regarding the interventions, during the day shift most of the face-to-face interventions were psychosocial, while the rest were attended only by the HSW when the need for psychological aid was not detected. Although most of the interventions were EOL, some of them involved supportive communication with the relatives of a deceased person. This is an example of a situation initially not contemplated that the program ended up covering and adapting to. Most EOL interventions took place on the same day or the day before the decease. Also, most of the relatives could come to the hospital in person to bid farewell to their loved ones. Sadly, due to the unpredictability and severity of COVID-19, some relatives arrived after the death of their loved one. In other cases, the team did not even have time to contact them because of the sudden death of the patient while being admitted to the hospital.

Regarding the staff's experience, we highlight the reported emotional impact due to the nature of the work and the fact that it was carried out in unusual conditions (49). Working and sharing as a team, or being on shifts that were not too long was considered by the team as ways to mitigate the perceived impact.

As stated, to our knowledge no other face-to-face EOL programs have been formally presented in the COVID-19 context. Only one similar program during the COVID-19 pandemic was found in a phone-based format (50), and although no quantitative efficacy assessment was reported they identified several useful roles CP can play in this scenario (29). Other EOL programs in ICU prior to the pandemic were found (45), using different approaches involving multidisciplinary family meetings, communication facilitators, and collaborations with palliative care professionals. Most of the interventions were not found to be effective because they were not guided by families' perceptions and needs, an aspect we made sure to incorporate whenever possible.

## Strengths and Limitations

Among the strengths of this study, the face-to-face nature of our program, in the midst of the high-restriction pandemic context, constitutes its main effectiveness. Another strength is its feasibility and ability to adapt and grow in an unstable context and chaotic environments. We also value its multidisciplinarity. On creating teams formed of HSW and CP in coordination with the HCT, we managed to provide a combined interdisciplinary intervention.

Nevertheless, it has some limitations. The first one is the lack of efficacy assessment of PGD prevention, basically due to ethical considerations. In further studies, outcomes measured to assess the efficacy of intervention should be taken into account, in order to evaluate the potential impact of the interventions on both families and professionals. Regarding the emotional impact on the team, we believe a higher level of rotation could help to mitigate it. Finally, to create a greater team spirit and to cover night shifts with psychological aid, the shifts should be unified for all professional categories.

## Conclusion

Structured programs addressing EOL situations and taking care of families and patients during the death and mourning processes should be a priority to prevent PGD and other associated complications. The COVID-19 pandemic placed the health system in a critical situation, where more programs responding to patients' psychosocial needs and those of their families are required.

## Data Availability Statement

The datasets used and analyzed during the current study will be made available by the corresponding author upon reasonable request

## Ethics Statement

The studies involving human participants were reviewed and approved by Clinical Research Ethics Committee (CEIm) of Vall d'Hebron University Hospital. Written informed consent for participation was not required for this study in accordance with the national legislation and the institutional requirements.

## Author Contributions

All the authors have contributed to the intervention, design, acquisition, analysis, interpretation, and/or review of the work and agree to be accountable for all aspects of the work, ensuring that questions related to the accuracy or integrity of any part of the work are appropriately investigated and resolved.

## Conflict of Interest

JR-Q was on the speakers' bureau and/or acted as consultant for Eli-Lilly, Janssen-Cilag, Novartis, Shire, Takeda, Bial, Shionogui, Lundbeck, Almirall, Braingaze, Sincrolab, Medice and Rubió in the last 5 years. He also received travel awards (air tickets + hotel) for taking part in psychiatric meetings from Janssen-Cilag, Rubió, Shire, Takeda, Shionogui, Bial, Medice and Eli- Lilly. The Department of Psychiatry chaired by him received unrestricted educational and research support from the following companies in the last 5 years: Eli-Lilly, Lundbeck, Janssen- Cilag, Actelion, Shire, Ferrer, Oryzon, Roche, Psious, and Rubió. The remaining authors declare that the research was conducted in the absence of any commercial or financial relationships that could be construed as a potential conflict of interest.
